# Risk factors for mortality in patients with hypoplastic left heart syndrome after the Norwood procedure

**DOI:** 10.1093/icvts/ivad127

**Published:** 2023-08-07

**Authors:** Sabina Selenius, Johanna Ilvesvuo, Hanna Ruotsalainen, Ilkka Mattila, Tommi Pätilä, Emmi Helle, Tiina Ojala

**Affiliations:** New Children’s Hospital Pediatric Research Center, Helsinki University Hospital, Helsinki, Finland; Stem Cells and Metabolism Research Program, Faculty of Medicine, University of Helsinki, Helsinki, Finland; Department of Obstetrics and Gynecology, Women’s Hospital, Helsinki University Hospital and University of Helsinki, Helsinki, Finland; Department of Pediatrics, Kuopio University Hospital, Kuopio, Finland; Department of Cardiac and Transplantation Surgery, Children’s Hospital, University Hospital of Helsinki and University of Helsinki, Helsinki, Finland; Department of Cardiac and Transplantation Surgery, Children’s Hospital, University Hospital of Helsinki and University of Helsinki, Helsinki, Finland; New Children’s Hospital Pediatric Research Center, Helsinki University Hospital, Helsinki, Finland; Stem Cells and Metabolism Research Program, Faculty of Medicine, University of Helsinki, Helsinki, Finland; New Children’s Hospital Pediatric Research Center, Helsinki University Hospital, Helsinki, Finland

**Keywords:** Norwood procedure, Hypoplastic left heart syndrome, Congenital, Mortality, Length of hospital stay

## Abstract

**OBJECTIVES:**

Several studies have reported mortality risk factors associated with hypoplastic left heart syndrome (HLHS). However, these data are ambiguous and mainly focused on the independent effects of these factors. We examined both the independent and the cumulative effects of preoperative risk factors for poor outcome in patients undergoing the Norwood procedure. Moreover, we studied the risk factors associated with prolonged initial hospital stays in these patients.

**METHODS:**

We performed a retrospective national 18-year observational study of preoperative risk factors for 1 year, as well as total follow-up mortality or need for transplant in patients with HLHS (N = 99) born in Finland between 1 January 2004 and 31 December 2021.

**RESULTS:**

Overall, one-year survival was 85.6%. In a multivariable analysis, having a major extracardiac anomaly and being small for gestational age were significant predictors of one-year mortality or the need for a transplant. Aortic atresia was a predictor of total follow-up mortality. An analysis of the cumulative effect indicated that the presence of 2 risk factors was associated with higher mortality.

**CONCLUSIONS:**

HLHS remains the defect with the highest procedural risks for mortality in paediatric cardiac surgery. From a prognostic point of view, recognition of independent preoperative risk factors as well as the cumulative effect of risk factors for mortality is essential.

The results of this study were presented orally at the 55th Annual Meeting of the Association for European Paediatric and Congenital Cardiology, Geneva, Switzerland, 28 May 2022.

**Figure:**
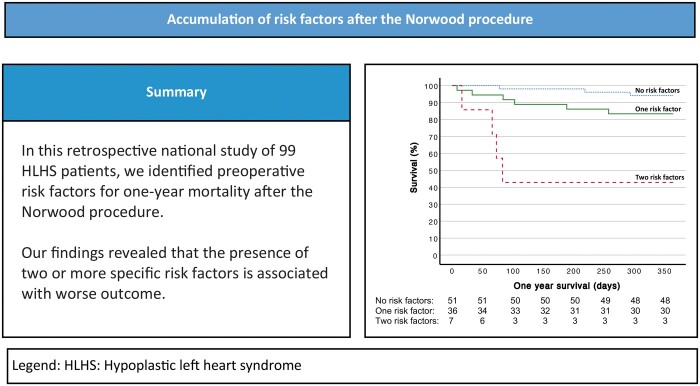

## INTRODUCTION

Hypoplastic left heart syndrome (HLHS) is characterized by atrioventricular and ventriculoatrial concordance with mitral and/or aortic stenosis or atresia and left ventricular hypoplasia [1]. The total and live birth prevalences of HLHS in Finland are 3.66 and 1.78 per 10 000 births, respectively [2]. HLHS is treated with a three-stage palliation, starting with the Norwood procedure in the first week of life, followed by the Glenn procedure, usually performed at 4–6 months of age and culminating in the Fontan procedure at 3–4 years of age.

Several preoperative and anatomical findings have been identified as risk factors for mortality and morbidity in HLHS [3]. These risk factors can either be directly life threatening (e.g. patency of the atrial septum and ductus arteriosus) or more prognostic in nature [3]. Previous studies have focused mainly on the independent effects of these risk factors, although a poor outcome is more likely multifactorial [4–9]. Understanding the cumulative effect of these risk features is important in clinical decision making. Our goal was to investigate how the combination of preoperative risk factors predicted outcome in patients undergoing the Norwood procedure.

## PATIENTS AND METHODS

### Data sources

We performed a retrospective national 18-year observational study of preoperative risk factors for 1 year and total follow-up mortality or the need for transplant in all patients with HLHS born in Finland between 1 January 2004 and 31 December 2021 who underwent the Norwood procedure. The initial care of all patients with HLHS receiving active care in Finland is centralized at New Children’s Hospital, Helsinki. The data were collected from national registers maintained by the Finnish Institute for Health and Welfare and by Statistics Finland, including the Care Register for Health Care, the Medical Birth Register, the Register of Congenital Malformations, the Cause-of-Death Register and hospital medical records.

### Ethical approval

The ethics committee of Helsinki University Hospital approved the study (HUS/1938/2016). The Finnish Institute for Health and Welfare authorized the use of the health register data in this study, as required by national data protection legislation. Participants were not contacted; no informed consent was required.

### Study population

Between 2004 and 2021, a total of 107 patients underwent the Norwood procedure in Finland, of which a total of 99 patients (93%) were diagnosed with HLHS. Patients with other univentricular heart defects undergoing the Norwood procedure [*N* = 8 (7%)] were excluded. HLHS was defined by the presence of an underdeveloped left ventricle and aortic stenosis/atresia and/or mitral stenosis/atresia.

### Data characteristics

The maternal, prenatal, anatomical and operative characteristics of patients are listed in Table 1[TQ1]. Maternal body mass index was calculated based on height and pregestational weight recorded at the first prenatal visit. A body mass index above 30 kg/m^2^ was considered obese. Small for gestational age (SGA) was defined as a birthweight below the 10th percentile of the gestational age. Extracardiac anomalies (ECA) included chromosomal anomalies and major extracardiac structural malformations defined according to EUROCAT (European network of population-based registers for the epidemiological surveillance of congenital anomalies) guidelines. The ECA present in our cohort are listed in Supplemental Table 1.

**Table 1:** Characteristics

Variable	Cohort (N = 99)
Infant characteristics	
Male sex, %	62 (61/99)
Birthweight, grams [SD]	3480 [486]
Gestational age, weeks [SD]	39.5 [1.3]
Small for gestational age, %	5 (5/99)
Structural characteristics	
Major extracardiac anomaly, %	12 (12/99)
Aortic atresia, %	36 (36/99)
Restrictive foramen ovale, %	33 (33/99)
Total or partial pulmonary vein drainage, %	5 (5/99)
Maternal characteristics	
Maternal age, years [SD]	30.9 [6.0]
Maternal BMI, kg/m^2^ (IQR)	25.7 (22.4–28.3)
Operative characteristics	
Blalock-Taussig shunt, %	22 (20/93)
Age at Norwood, days (IQR)	6.0 (5–8)
Weight at Norwood, grams[SD]	3510 [476]
Aorta cross-clamp time, min (IQR)	65.5 (46–95)

BMI: body mass index; IQR: interquartile range; SD: standard deviation.

The diagnostic criteria for restrictive foramen ovale were (1) a diameter of < 2.5 mm; and (2) a mean interatrial gradient of > 4 mmHg during the entire cardiac cycle. Certain contraindications for active care in this patient group are present at our institute. These include an intact atrial septum, moderate to severe tricuspid valve regurgitation and/or poor preoperative ventricular function. Patients with total or partial anomalous pulmonary vein drainage (TAPVD/PAPVD) (*N* = 5) were excluded from the multivariable analysis because TAPVD is a known risk factor for death and has been an exclusion criterion for surgery at our institute since 2005.

In addition, parents are given the option of choosing compassionate care, and infants who received compassionate care were not included in our analysis.

Outcome variables included (1) death or a transplant within 1 year of birth; (2) death or a transplant during the total follow-up time from birth until 31 December 2021; and (3) length of initial stay (LOS) in the hospital for first-year survivors. Initial LOS was defined as hospitalization days from birth until first discharge home or to another hospital.

### Statistical analyses

Univariable analysis of independent risk factors for death or a transplant was carried out using the χ^2^ test or Fisher’s test when appropriate. Continuous variables were analysed using an independent sample *t*-test for normally distributed variables, and the Mann–Whitney U-test was used for non-normally distributed variables. The Kolmogorov-Smirnov and Shapiro-Wilk tests were used to determine normal distribution. Continuous variables are reported as means and standard deviation for normally distributed data, and medians and interquartile range are reported for non-normally distributed data. Factors associated with mortality with a *P*-value < 0.1 in the univariable analysis were included in the multivariable model. The multivariable model was analysed using Cox regression. All the tests were two-sided.

Analysis of multiple risk factors for death was conducted using Cox regression analysis. The results were visualized using Kaplan–Meier survival curves.

Initial LOS was positively skewed and was log transformed before analysis. The correlation with LOS was carried out using the two-sample *t*-test for categorical variables and the Pearson correlation for continuous variables in the univariable analysis. One extreme outlier with an initial LOS of 477 days was excluded from the analysis. Factors associated with LOS in the univariable analysis with a *P*-value < 0.1 were included in the multivariable linear model. Statistical analyses were performed using IBM SPSS Statistics 27 for Windows (IBM Corp., Armonk, NY, USA). All the tests were two-sided. *P*-values < 0.05 were considered statistically significant.

## RESULTS

Of 99 patients with HLHS, 14 died and 2 received a heart transplant within 1 year of birth. During the total follow-up period (median 9.4 years, range 6 days–17.9 years), 17 patients died and 4 patients received a heart transplant. Overall, the one-year survival was 85.6%, and total follow-up survival was 82.8%. Deaths within 30 days of the Norwood procedure were 4%.

### Univariable analysis for death or transplant

Results of the univariable analysis are presented in Supplemental Table 2. Small for gestational age was a significant predictor of both one-year mortality and total follow-up mortality. The presence of TAPVD/PAPVD was a significant independent predictor of one-year mortality, and aortic atresia (AA) was a significant independent predictor of total follow-up mortality.

### Multivariable analysis for death or transplant

For the one-year follow-up, birthweight, AA, SGA and major ECA had a *P*-value <0.1 in the univariable analysis and were included in the Cox regression analysis. Patients with TAPVD/PAPVD (*N* = 5) were excluded.

In the multivariable analysis, major ECA [hazard ratio (HR) = 4.03, 95% confidence interval (CI) 1.08–15.07) and SGA (HR = 10.55, 95% CI 1.87–59.48), were significant predictors of one-year mortality (Table 2). For the total follow-up, birthweight, AA and SGA had a *P*-value <0.1 in the univariable analysis and were included in the Cox regression analysis. In the multivariable analysis, AA (HR = 4.36, 95% CI 1.45–12.56) was a significant predictor of follow-up mortality (Table 3).

**Table 2:** Cox regression analysis of risk factors for one-year mortality or transplant

Variable	Hazard ratio	95% CI	*P*-value
Aortic atresia	2.23	0.71–7.13	0.17
Major extracardiac anomaly	4.03	1.08–15.07	0.038
Birthweight	1.00	0.999–1.002	0.83
Small for gestational age	10.55	1.87–59.48	0.008

**Table 3:** Cox regression analysis of risk factors for follow-up mortality or transplant

Variable	Hazard ratio	95% CI	*P*-value
Aortic atresia	4.26	1.45–12.56	0.01
Birthweight	1.00	0.999–1.001	0.96
Small for gestational age	11.60	0.80–167.6	0.07

### Cox regression of multiple risk factors

We next estimated how the presence of more than one risk factor, including AA, SGA and the presence of major ECA, affected mortality. The patients (*N* = 94) were divided into 3 groups based on the number of risk factors. The patients in group 1 had no risk factors; the patients in group 2 had 1 risk factor and the patients in group 3 had 2 risk factors. No patients had all 3 risk factors. The one-year mortality was 5/51 (5.9%) in group 1, 6/36 (16.7%) in group 2 and 4/7 (57.1%) in group 3.

**Figure 1:**
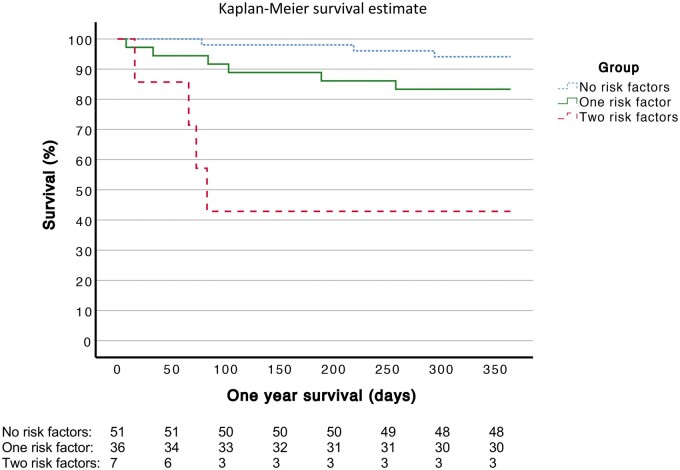
Kaplan–Meier analysis for one-year survival of patients with and without accumulation of risk factors.

The presence of 2 risk factors was associated with higher one-year mortality or the need for a transplant compared with patients with no risk factors (*P ≤* 0.001, HR 15.98, 95% CI 3.54–72.17) (Table 4; Fig. 1).

**Table 4:** Cox regression analysis of multiple risk factors

Variable	Hazard ratio	95% CI	*P*-value
No risk factors			
One risk factor	3.03	0.76–12.13	0.12
Two risk factors	15.98	3.54–72.17	<0.001

### Risk factors associated with prolonged length of stay

The overall median initial LOS of the survivors was 34 (interquartile range 18–252) days. The results of the univariable analysis for LOS are presented in Supplemental Table 3.

Aortic atresia, the shunt type and SGA were included in the analysis. According to the multivariable linear model, SGA (adjusted mean difference 0.30, 95% CI 0.19–1.13, *P* = 0.006) was associated with longer initial hospital stay (Table 5).

**Table 5:** Multivariable linear model of length of hospital stay

Variable	Odds ratio	95% CI	*P*-value
Small for gestational age	0.30	0.19–1.13	0.006
Aortic atresia	0.19	−0.010–0.21	0.07
Blalock-Taussig shunt	0.19	−0.013–0.25	0.08

Length of hospital stay was log-transformed for statistical analysis.

## DISCUSSION

In this national 18-year cohort, we showed that the presence of SGA and major ECA was associated with poor outcomes during the first year of life. Most importantly, patients with 2 risk factors had a particularly poor prognosis, with survival of less than 3 months among the deceased members of the group. These results are important to consider when deciding treatment strategies in patients with HLHS and highlight the need for enhanced postoperative monitoring of high-risk patients.

Our findings demonstrate that major ECA and SGA are independent predictors of a poor outcome within a year of birth and after the Norwood procedure. Additionally, AA was identified as a significant predictor of a poor outcome during the total follow-up period. This outcome may be attributed to potential coronary hypoperfusion before the Glenn procedure, residual issues with the anastomosis and increased risk of native aortic root thrombus, which could contribute to adverse outcomes in patients with AA.

Previous studies have shown these risk factors to be predictors of one-year mortality, operative mortality and hospital mortality [6, 10–13]. Our results also confirm the results that patients with SGA had a higher risk of poor outcome than patients who were normal or large for gestational age. Previous studies have found that prematurity and birthweight under 2500 grams are risk factors for death [8]. Prematurity, low birthweight and low weight at the time of the Norwood procedure were not independent risk factors for a poor outcome in the current study. This result may be due to the limited sample size: Only 3 patients were born prematurely (gestational weeks < 37), and only 1 patient had a birthweight below 2 500 grams. It has been shown that the right ventricle-pulmonary artery (RVPA) shunt is associated with higher transplant-free survival the first year than the Blalock-Taussig shunt, but also with a higher incidence of unintended interventions and complications [14]. In our study, the difference did not reach statistical significance; however, the burden of other risk factors may be affected by the type of shunt being used.

Interestingly, most deaths in our study occurred after the 30-day postoperative period, underlining the importance of sufficiently long follow-up time when assessing the outcome in this patient group.

Previous studies have reported ambiguous results concerning the role of individual risk factors for Norwood-associated deaths, with some studies showing no association between major ECA, SGA, AA and death [8, 15]. Other studies have identified other predictors of death, such as low birthweight, restrictive foramen ovale and gestational age [7, 8, 16]. This ambiguity could in part be due to the effect of one risk factor being enhanced in the presence of other risk factors. In our study, the presence of 2 risk factors was associated with increased one-year mortality or the need for a transplant. All patients who died in this group died by 3 months of age, which is likely due to the severity of the defect and/or the comorbidities. This finding highlights the first months as critical for survival and the need for enhanced postoperative monitoring of high-risk patients.

Only being SGA was associated with prolonged initial LOS in our study. Other risk factors might be associated with prolonged overall hospital stay, because we only looked at the initial hospital stay at the New Children’s Hospital before discharge to other hospitals.

The strengths of this study include the mandatory, high-quality Finnish registries, which facilitated the collection of comprehensive data for our national cohort. Initial care of patients with HLHS in Finland is centralized at the New Children’s Hospital in Helsinki. Thus, the standard of care is similar for every patient.

Limitations in our study include a small sample size, so the results should be interpreted with caution. Studies on larger cohorts are needed to confirm our findings. It is important to point out that, in this cohort, those with an intact atrial septum, moderate tricuspid valve regurgitation and/or poor ventricular function preoperatively were excluded from operative treatment according to the national guidelines. Furthermore, it was not possible to review a few of the patient records after the patients were discharged to other hospitals.

## CONCLUSION

Our study demonstrates SGA and extracardiac malformations as predictors of worse outcomes after the Norwood procedure. In addition, the accumulation of risk factors was associated with an increased risk of poor outcome. Patients with more than 1 risk factor should be considered for enhanced postoperative monitoring. An important implication of our findings is the need for future efforts to improve the outcome through possible preoperative interventions in high-risk patients. These findings are also important to consider when evaluating patients for surgical treatment.

## Supplementary Material

ivad127_Supplementary_Data

## Data Availability

The data underlying the current study are available on reasonable request from the corresponding author.

## Abbreviations

AA: Aortic atresia
ECA: Extracardiac anomaly
HLHS: Hypoplastic left heart syndrome
LOS: Length of stay
SGA: Small for gestational age
TAPVD/PAPVD: Total or partial anomalous pulmonary vein drainage
