# Supplementary material for: Risk factors for mortality in patients with hypoplastic left heart syndrome after the Norwood procedure
Source: Interdiscip Cardiovasc Thorac Surg. 2023 Aug 7;37(2):ivad127. doi: 10.1093/icvts/ivad127 (PMC10448988; doi:10.1093/icvts/ivad127)
Supplement: ivad127_Supplementary_Data [file ivad127_supplementary_data.docx]

**Supplemental tables**

| **Supplemental Table 1.** List of major extracardiac anomalies, including chromosomal anomalies and extracardiac malformations, included in the study. | |
| --- | --- |
| **Major extracardiac anomalies** |  |
| **Chromosomal anomalies** |  |
|  | 46X, i(X)(q10) Turner |
|  | Mosaicism, 46, i(X), 45X, turner |
|  | Interstitial microdeletion 16p13.11 |
|  | Duplication (1) (q21.1) |
|  | Kabuki syndrome |
| **Extracardiac malformations** |  |
|  | Congenital hypothyroidism, immature ganglions of colon |
|  | Hypospadias penile |
|  | Craniosynostosis |
|  | Micrognathia |
|  | Severe hypoplasia of hand |
|  | Esophageal atresia |
|  | Pyloric atresia |
|  | Retention of testis bilaterally |
|  | Rocker-bottom feet |
|  | Colon malrotation |

**Supplemental table 2:** Univariate analysis of one-year mortality and follow-up mortality.

| Variables | One-year mortality | | | Follow-up mortality | | |
| --- | --- | --- | --- | --- | --- | --- |
|  | Survivors (N=83) | Deceased (N=16) | P-value | Survivors (N=79) | Deceased (N=21) | P-value |
| Male sex (%) | 61.4% (51/83) | 62.5% (10/16) | 0.94 | 60.3% (47/78) | 66.7% (14/21) | 0.59 |
| Birthweight, grams | 3517.18 [49.48] | 3286.56 (155.19] | 0.082 | 3525.65 [457.29] | 3310.00 [560.64] | 0.071 |
|  |  |  |  |  |  |  |
|  |  |  |  |  |  |  |
| Gestational age, weeks | 39.6 [0.14] | 39.2 [0.33] | 0.19 | 39.6 [1.29] | 39.2 [1.22] | 0.23 |
| SGA, % | 1.2% (1/83) | 25.0% (4/16) | 0.002 | 1.3% (1/78) | 19.0% (4/21) | 0.007 |
|  |  |  |  |  |  |  |
|  |  |  |  |  |  |  |
| Major ECA, % | 9.6% (8/83) | 25.0% (4/16) | 0.10 | 10.3% (8/78) | 33.3% (4/21) | 0.28 |
| AA, % | 32.5% (27/83) | 56.3% (9/16) | 0.071 | 28.2% (22/78) | 66.7% (14/21) | 0.001 |
|  |  |  |  |  |  |  |
|  |  |  |  |  |  |  |
| Restrictive foramen ovale, % | 31.3% (26/83) | 43.8% (7/16) | 0.33 | 32.1% (25/78) | 38.1% (8/21) | 0.60 |
| TAPVD/PAPVD, % | 2.4% (2/83) | 18.8% (3/16) | 0.029 | 2.6% (2/78) | 14.3% (3/21) | 0.063 |
|  |  |  |  |  |  |  |
|  |  |  |  |  |  |  |
| Maternal age (years, [SD]) | 30.9 [0.675] | 31.4 [1.46] | 0.76 | 30.8 [6.01] | 31.4 [6.27] | 0.72 |
| Maternal BMI (kg/m2, (IQR)) | 25.9 (22.5 – 28.3) | 25.5 (21.75 – 27.7) | 0.60 | 26.4 (23.1 - 29) | 25 (22.2 – 28.4) | 0.64 |
| BT-shunt (%) | 19.2% (15/78) | 33.3% (5/15) | 0.30 | 20.5% (15/73) | 25.0% (5/20) | 0.76 |
| Age at Norwood (days, (IQR)) | 6 (5 – 8) | 6 (4-7) | 0.15 | 6 (5 – 8) | 6 (4.5 – 7) | 0.17 |
| Weight at Norwood (grams, [SD]) | 3542.2 [0.049] | 3323.6 [0.17] | 0.11 | 3547.6 [0.454] | 3359.5 [0.546] | 0.12 |
| Aorta cross clamp time (minutes, (IQR)) | 66 (48.5 - 95) | 65 (39-91) | 0.82 | 65.5 (50 – 91.3) | 86 (52.5 – 96.5) | 0.96 |

**Supplemental table 3:** Univariate analysis of initial length of hospital stay in survivors

|  | | |
| --- | --- | --- |
| Variable | LOS in days |  |
| Categorical variables | **Median (IQR)** | **P-value** |
| Sex |  | 0.91 |
| Male (N =50) | 34 (26-47) |  |
| Female (N=32) | 33.5 (25-52) |  |
| Restrictive foramen ovale |  | 0.49 |
| Yes (N=56) | 35.5 (18–136) |  |
| No (N=26) | 32.5 (18–252) |  |
| AA |  | 0.03 |
| Yes (N=26) | 39.5 (23–252) |  |
| No (N=56) | 32 (18–142) |  |
| Major ECA |  | 0.26 |
| Yes (N=8) | 34 (21–172) |  |
| No (N=74) | 34 (18–252) |  |
| TAPVD/PAPVD |  | 0.76 |
| Yes (N=1) | 34 (34) |  |
| No (N=74) | 34 (18–252) |  |
| SGA |  | <0.001 |
| Yes (N=1) | 252 (252) |  |
| No (N=81) | 34 (18-172) |  |
| Shunt type |  | 0.015 |
| BT-shunt (N=15) | 35 (26-252) |  |
| RV-PA (N=62) | 32 (18-172) |  |
| Continuous variables | **Pearson correlation** | **P-value** |
| Maternal BMI (kg/m2) | 0.13 | 0.30 |
| Maternal age (years) | -0.002 | 0.99 |
| Birthweight (grams) | 0.002 | 0.99 |
| Gestational age (weeks) | -0.09 | 0.99 |
| Age at Norwood (days) | 0.14 | 0.21 |
| Weight at Norwood (grams) | -0.04 | 0.72 |
| Aorta cross clamp time (minutes) | 0.10 | 0.45 |
